# Supplementary material for: Patterns of Intron Gain and Loss in Fungi
Source: PLoS Biol. 2004 Nov 30;2(12):e422. doi: 10.1371/journal.pbio.0020422 (PMC532390; doi:10.1371/journal.pbio.0020422)
Supplement: Table S1 — Also available at http://genes.mit.edu/NielsenEtAl/. (4.3 MB ZIP). [file pbio.0020422.st001.zip › NielsenEtAl/html/909.html]

AN5592.1.NCU06067.1.MG09697.1.FG10511.1


```
 CLUSTAL W (1.82) Multiple Sequence Alignments - Introns Inserted


Sequence 1: NCU06067.1	970 aa
Sequence 2: MG09697.1	1127 aa
Sequence 3: FG10511.1	981 aa
Sequence 4: AN5592.1	913 aa
Alignment Length: 1153 aa
Number Identitical Residues: 283 aa
Alignment Score (without introns) 16357


MG09697.1 	MNQASRR2QPGLNAPRDTKLAGSVQSNDVNQQPLLPTPAPKPP0DQSLPGPSRRSASENI
NCU06067.1	-------~-----------------------------------~----------------
FG10511.1 	-------~-----------------------------------~----------------
AN5592.1  	-------~-----------------------------------~----------------
          	                                                            

MG09697.1 	GVGHPTCPVAFTPPNALAMAYAPLLRTNTTPIFPSSTGGVARLQGTMGSTGPAVGIPPLR
NCU06067.1	----------------------------------------------MSLSN------AAR
FG10511.1 	----------------------------------------------MNHAP--------R
AN5592.1  	------------------------------------------------------------
          	                                                            

MG09697.1 	ASQLSGTTLHN-STTSLSSLASASTVVPAQNGGQVVATSNIINQKADASRSLYQICMSLK
NCU06067.1	ASQLSGSTAFN-SSTSLSSLTSSATAVTPVNG-QVVATTNIINQRADASRSLYQICLSLK
FG10511.1 	ASQLSGSTAYTGSSASLSSLAT-ATTITPTNGGPVHATANIINQKADASRSLYQICISLK
AN5592.1  	-----------------------MADTAGLNPGGPVAEDNIINRRGNEG--IYQSCVNLK
          	                        :  .  * .   *  ****::.: .  :** *:.**

MG09697.1 	QRLAQVPGFEEYMSLMDQWEAEDPEGGIVESVWKLLRMGTPLIVIFNLLKPENPMSFDPT
NCU06067.1	QRLAQVPGFEGYLEQL-QREQENGHDGPVEALWKLLRTGLPLLTIYNALQPEKPLHVDQR
FG10511.1 	QRLAQVPGFGPYLDELDPTDP-------VDPLWNLFRSGYPLLLIYNALRPNEELKVDDS
AN5592.1  	KRLAEVPGFEPYLREMEEEDLAQGNSDPVASLWNYLRHGYPLLAIYNASDPGAPLEIDTS
          	:***:****  *:  :.  :  .  .. * .:*: :* * **: *:*   *   : .*  

MG09697.1 	P---DAKKAKMYIYKFAEQCKNELG--IEDIFTISDLLKNDTTGFVK0VTTVVNQVLDIA
NCU06067.1	PNETEARRSKRAIMEFVKACLSDLDPPVTECFIVADLTGEDTTGFVK0VTSVINYVLDLA
FG10511.1 	SSANEAKKSKIAIFKFVQACMKELEIPSTQSFVITDLMGTDTSGFVK0VTQVVNYVLDRA
AN5592.1  	KVP-EARRPKAATFKFLQAAIQEMAFPQQECFLITDLYGENTIGFTK~VIKMVNRVLDIL
          	    :*::.*    :* : . .::  .  : * ::**   :* **.* *  ::* ***  

MG09697.1 	QTRGMLIQQQPYPEDDMPDSGKSKMSYRDYIVRELVDTERKYVQDLENLHDLKKALTERG
NCU06067.1	EKRGLLLQTLPYPEDDITQPG-SKMTHRDYIVRELVDTERKYVQDLENLHDLKRTLEHRS
FG10511.1 	EERGYLMQAQPDVESNEPTGG--QMTYRDHIIRELVDTERKYVQDLENLHDLKKTLEQQG
AN5592.1  	EIQGQLKKP-SDTAMAAPAAG-RKLTKREHILKELLETERDYVHHLQNLQALKKELEDTG
          	: :* * :  .      .  *  ::: *::*::**::***.**:.*:**: **: * . .

MG09697.1 	IVTGDVAYQIFGNIDAILDFQRRFLIRVETTNSMPGEVQQWGSPFVAMEASFDPIYTPFI
NCU06067.1	VIPGEFIHDIFLNINAILDHQRKFLIRVETTNSMPQARQEWGSLFVTAEENFG-IYQPFI
FG10511.1 	EIPGDTLHQIFFNINAILDFQRRFLIRVETTNSMPALNQRWGAPFVHYEDALIDIYQPFI
AN5592.1  	ALTGDASHQIFLNLNNLLDFSQRFLIRLEQHYARPEEQQNWGELFIQHEEAFR-QYEPFI
          	 :.*:  ::** *:: :**..::****:*   : *   *.**  *:  *  :   * ***

MG09697.1 	ANQRKAALIAQSEFDKIKSIE-----HPVACDFNTLDGFLLKPMQRLVKYPLLLK0DLLK
NCU06067.1	ANQRKAAQVATQVFDKIQEAG-----HPVACDFNTLDGFLLKPMQRLVKYPLLLK0DLLK
FG10511.1 	ANQRKAAQVANQVFDKIQRSS-----HPVAADYNTLDGFLLKPMQRLVKYPLLLK0DLNK
AN5592.1  	ANQMRCDKTCQKEWDKIQAAPRSPDLQQMVAQPATLNGFFVKPFQRLTKYPLMLS0ELRK
          	*** :.   . . :***:    :.. : :..:  **:**::**:***.****:*. :* *

MG09697.1 	KSEDDKVKDDLSLGIAAAERTLQKANEAVDRDLLDDALEDLKTRVDDWKNHQVDQFGSLL
NCU06067.1	KSEDEHTKEDLAAGIAAAERVLMKANSAVDKNILEEALQDLIHRVDDWKSHKVDNFGSLL
FG10511.1 	KTEDDEVKMDLTTGCEAAERVLSKANEAVNRDLLDEAVEDLTSRVEDWKSHKVEQFGKLL
AN5592.1  	QIEDPDLQADISRAIDSIQSVLDAANDAIDKEQLAAAFVELDERVDDWKALKIETFGELL
          	: ** . : *:: .  : : .*  **.*:::: *  *. :*  **:***  ::: **.**

MG09697.1 	LHGVHTVLTGKS--ESERD0YEIYLFECILLCCKEISSTKSKDKKDKTKSTGPKIRNKGA
NCU06067.1	LHGVYTVITGKS--EQEKD0YEIYLFENILLCCKELTTTKAKDKKDKTRSSVPKLRNKFA
FG10511.1 	LHGVYGVITGKT--DQEKD0YEIYLFESILLCCKEISSSKSKDKKDKLRSGGPKARNKSA
AN5592.1  	RFGTFTVIKNDNNKDSERE0YHIYLFERILLCCKDINPNKQKSRLVGGSKDKPNTSKGKP
          	 .*.. *:......:.*:: *.***** ******::...* *.:     .  *:  :  .

MG09697.1 	KLQLKGRIFMTNVTEVLSFAKPG~SYTVQIWWKGDPG-VENFIIKFQNEEMMKKWAAGLD
NCU06067.1	KLQLKGRIFMTNVTDVVAISKPG1SYTVQIWWKGDPG-VENFMIKFQTEEMMKKWAAGLE
FG10511.1 	KLQLKGRIFMTNVTDIVSFTKPG1SHSVQIWWKGDPG-VENFIIKFLNEETLKKWVNTLE
AN5592.1  	RLVLKGRIYMANVTDIVWLQKPG1SYRIQIFWKGDPGVVDNFIIRYQNEDTMRKWYKDIN
          	:* *****:*:***::: : *** *: :**:****** *:**:*:: .*: ::**   ::

MG09697.1 	QQRKASG-SQSATSPDQPTAEFAWMRSQ-SAVENPYAQTQPEEEEEYEFGAIPQQSQQYQ
NCU06067.1	EQRKQNT-QAAPVAPDQAPPNFAWLASQGNGLENPYKDEEEDED-------IYNAAAPTV
FG10511.1 	TQRKHNV-PRQSTNSDTLSTDFAWTRDQVAGLENPYLKENDDEDDEDIGPATAPAGFPGV
AN5592.1  	TQREIQAEQRSARNTGTSDSEFTYMKSL-SNIPNPYQQEYDVEE---------QSTKEAA
          	 **: . .   .  ..   .:*::  .    : *** .    *:                

MG09697.1 	AMAGTM-PRMNSTASIRGRSQTGESSQSIASIARGPPPRFPLPQPPQPLSVQTQLQGG-N
NCU06067.1	AMPGTM-PRNGSSTSLRQRSAT---SDSLAGMARAPPPRFPLPQAPAPLNVGLQP-----
FG10511.1 	THPMSLGPRTASSNNLRARAGTGESSASLAGMVRAPPPRFPLPAPPGSLSLQTQPNGA-H
AN5592.1  	FFSEFPMSRNASSTSLRTRSATGGSGSSGPPLSTSRP-RYP-AMPDSTLSVHTQFPGGSM
          	  .    .*  *: .:* *: *. :. * . :  . * *:* . .  .*.:  *  ..: 

MG09697.1 	SPGFRPGDSYFSPIGESPVSSRTSTASNVFPGSAGYGSFKGVTGQPYPGMWDGPIVDQNR
NCU06067.1	SPAVRAGESYFSPVAESPASSRTSTASGIFAG-AGSGYPFPKTGTPQG--WQ---EDSNR
FG10511.1 	SPSAWAGDSYFSPVTESPASTRTSTTSGMFST-PQYGFPKSATPNPQQ--WED--ANGNR
AN5592.1  	SPGERNGNSYFSPT----ESARSSSQS--------AGYPYNRQVTPVTP-WGD---DNNR
          	**.   *:*****      *:*:*: *         *        *    * .   : **

MG09697.1 	YTAPAMPRAPSRDGPSPVNAYGMSGANGRNPRGPSMPVMASHSAQGASQQSRSRSYSTPD
NCU06067.1	YTAPAMPRAPSRDGPSPG--------TGRNPRGPSMPVMP-RGDNSQFQQARSRSYSTPD
FG10511.1 	YTAPAMPRAPSRDGPSPN--------PARNPRGPSLPAMSSSSQAALAAQQRNRSYSTPD
AN5592.1  	YTAPALSRATSRDGSNSGYFNG-APPNGRSAQRPSLPPMSGSNQSSNSASQRMRSASSPD
          	*****:.**.****...    . : . .*..: **:* *.. .  .   . * ** *:**

MG09697.1 	IQGQGPPGMRRTPGGSQSNVP--AVPGIPAHLHAAHERHDSNIPRSNTGSPALSLRNDLP
NCU06067.1	IAGQAAARIN------QTGVP--AVPGIPAHLT--HARHDSNIPRSNTGSPAN----DLP
FG10511.1 	INGPGMPRTRQP---SHGNIP--AVPGIPQHLH---PGHNPNLSRDQTGSPRN----DQP
AN5592.1  	IHHHNPESRRYMGVHTMQTVDNVPVPPIPAHMAN----MKAPVNRSQNNSPTN---QSLP
          	*        .     :   : . .** ** *:       .. : *.:..**     .. *

MG09697.1 	MRSNTQSPGVQRDRLMQQGGGYSGGTLAQFPMQPVYP---RGGTPNPPGSGHTPAPLN-L
NCU06067.1	LRTQTGSPGRERK--------NYGGQLNQFPPQPIRQGTPGSVTGFPPPSGPPPPGMAPL
FG10511.1 	TRAQTNSPGAQRER-MHKPTGSVGGSMNHFPAQPVHP------RSITPGGGNQILRVD--
AN5592.1  	IRTNTSHAFHEPQY-------SDGRAAAPLSDQPTSP------LSHEPEE----------
          	 *::*  .  : .          *     :. **             *            

MG09697.1 	SADMG-RTVSPPLGTGTPQPPNTAGLMSPDQGGGIMPTQLKVKVNYDTSNYMTLVAAYNI
NCU06067.1	APTEGTRNITPSLGTAPIMSGQNP--LSP-TDDDMLPTQLKVKVHCDTGNYITLVVLYDI
FG10511.1 	AAAANSRTVSPALGTAT-MPPPSANPLSP---EIPLPTQLKVRVNCESGNYVTLVVAFNI
AN5592.1  	--------------------------------EPFMPTQLKAKVNFD-ENYVTLVISSNI
          	                                   :*****.:*: :  **:***   :*

MG09697.1 	TYRSLVDRIDAKLARFTNSSISRGNLRLRYRDEESDFVSIVCDEDVQMAFTDGRNGDRDM
NCU06067.1	TYEKLIDRIDNKLSRFTSSSIGGGNLRLRYRDEDGDFVMIESDDDIQIAISDWREGLRNP
FG10511.1 	TYQSLVDRIDAKLARFTTSSISKSMLKLRYRDEDGDYVAIEGDDDIQIAFMEWREGVRNM
AN5592.1  	GFRTLTDRVDAKLARFTNRSIGSKTVRLRYQDEDGDFVTIDSDEAVQLAFVEWKEQHREE
          	 :..* **:* **:***. **.   ::***:**:.*:* *  *: :*:*: : ::  *: 

MG09697.1 	YNG-GVGEVELFCVGIGGE
NCU06067.1	DS---AGEIDLYCEGELLE
FG10511.1 	YSG-GVGEIELFCVGDTA-
AN5592.1  	LARGQVGEIQLFCQPIEN-
          	   . .**::*:*
```
